# Incidence and outcomes of acute kidney injury after cardiac surgery using either criteria of the RIFLE classification

**DOI:** 10.1186/s12882-015-0066-9

**Published:** 2015-05-30

**Authors:** Marc-Gilbert Lagny, François Jouret, Jean-Noël Koch, Francine Blaffart, Anne-Françoise Donneau, Adelin Albert, Laurence Roediger, Jean-Marie Krzesinski, Jean-Olivier Defraigne

**Affiliations:** Division of Nephrology, University of Liège Hospital (ULg CHU), Avenue de l’Hôpital, 1, B-4000 Liège, Belgium; Division of Cardio-vascular and Thoracic Surgery, ULg CHU, Liège, Belgium; Department of Medical Informatics and Biostatistics, Public Health, ULg, Liège, Belgium; Division of Anaesthesiology, ULg CHU, Liège, Belgium

**Keywords:** Cardiac Surgery, Cardiopulmonary Bypass, Acute Kidney Injury, RIFLE, Serum Creatinine, Urine Output, Outcome, Mortality

## Abstract

**Background:**

Adult cardiac surgery is significantly associated with the development of acute kidney injury (AKI). Still, the incidence and outcomes of AKI vary according to its definition. Our retrospective monocentric study comparatively investigates the yield of RIFLE definition, which is based on the elevation of serum creatinine levels (SCr) or the reduction of urine output (UO), taking into account only one or both criteria. Pre- and per-operative risk factors for post-operative AKI were evaluated.

**Methods:**

All adult patients undergoing cardiac surgery, with or without cardiopulmonary bypass, from April 2008 to March 2009 were included. Clinical, biological and surgical features were recorded. Baseline serum creatinine was determined as its value on day 7 before surgery. Post-operative AKI was diagnosed and scored based upon the highest serum creatinine and/or the lowest urine output.

**Results:**

443 patients (Male/Female ratio, 2.3; median age, 69y) were included, with 221 (49.9 %) developing postoperative AKI. Elevated serum creatinine (AKI_SCr_) and oliguria (AKI_UO_) was observed in 9.7 % and 40.2 %, respectively. AKI patients had a significantly higher BMI and baseline SCr. In comparison to AKI_UO_, AKI_SCr_ mostly occurred in patients with co-morbidities, and was associated with an increased mortality at 1-year post surgery.

**Conclusions:**

The use of standard RIFLE definition of AKI in a cohort of 443 patients undergoing cardiac surgery resulted in an incidence reaching 50 %. Still, significant discrepancies were found between AKI_SCr_ and AKI_UO_ regarding the incidence and outcomes. In line with previous reports, our data questions the utility of urine output as a criterion for AKI diagnosis and management after cardiac surgery.

## Background

Adult cardiac surgery, with or without cardiopulmonary bypass (CPB), remains significantly associated with acute kidney injury (AKI). The incidence of post-surgery AKI varies from 5 to 42 % according to recent studies [1, 2]. In order to properly assess and compare the incidence and outcomes of AKI worldwide, the Acute Dialysis Quality Initiative Group proposed in 2004 a standard classification termed “RIFLE”, which stands for the acronym “Risk, Injury, Failure, Loss of kidney function and End stage kidney disease”, and is based on 2 criteria: serum creatinine levels (SCr) and urine output (UO) [3]. The main objectives of the present retrospective monocentric study were to (i) evaluate the incidence of AKI following cardiac surgery using the conventional RIFLE classification, and (ii) compare the yield of either RIFLE criteria to identify the patients developing AKI during the postoperative period. Next, we assessed the impact of AKI defined using either the full RIFLE classification or only one of either criterion on the length of in-ICU (intensive care unit) and in-hospital stays and on mortality at 1 year. Finally, we looked for the pre- and per-operative risk factors for post-operative AKI in our cohort.

## Method

### Patients

The present retrospective study was approved by the University Hospital of Liège Ethical Board (Ref. B70720108229). Data were collected from the database of University of Liège Hospital, and encompassed all adult patients who underwent heart surgery, including on- and off-pump coronary bypass grafting (CABG), isolated aortic or mitral valve reparation/replacement, or combined surgery (valve + CABG), from April 2008 to March 2009. Eight patients under chronic haemodialysis prior to surgery were excluded.

### Types of anaesthesia and surgery

Anaesthesia was classically performed through target-controlled infusions of propofol and remifentanil. Tranexamic acid was used as antifibrinolytic agent (2.5 g injected prior to the start of surgery and 2.5 g injected at the end of CPB). The volume of priming was 1600 mL consisting of 150 mL of Mannitol**®** 15 % and 1450 mL of Volulyte**®** 6 %, 1 g of tranexamic acid, as well as 5000 IU of unfractioned heparine. Minimal haemodilution level was 20 % haematocrit. Standard cannulation was carried out (i.e. into the ascending aorta for reinjection and single cannulation of the right auricle for On-pump CABG and aortic valve surgery, double venous cannulation for mitral valve surgery). CPB (roller pump) was initiated to maintain a flow between 2.4 L/m^2^/min and 3.2 L/m^2^/min. Most interventions were conducted using normothermia, with intermittent cold crystalloid cardioplegia. Blood discharged from the left ventricle was collected in the venous reservoir of the CPB. The pericardial blood was suctioned in a separate reservoir, treated through an autologous transfusion system and re-transfused into the CPB circuit or directly to the patient after neutralisation of heparin for On-pump CABG. For isolated valve or combined surgery, the pericardial blood was re-infused to the CPB’s venous reservoir.

### Clinical and biological variables

The following variables were analysed: gender (male vs female), age (years), body mass index (BMI, kg/m^2^), presence of high blood pressure (arterial pressure ≥140/90 mmHg) and/or treatment for high blood pressure (yes vs no), history of chronic obstructive pulmonary disease (COPD, yes vs no), angina pectoris (absence vs presence), myocardial infarction prior surgical intervention (yes vs no), diabetes mellitus (DM, yes vs no), emergency surgery (yes vs no), preoperative glomerular filtration rate (GFR, mL/min) and hemoglobin (g/dL), left ventricular ejection fraction < 60 % (LVEF < 60 %, yes vs no), surgical delay (days between the diagnostic coronarography and surgical intervention), type of operation (off-pump CABG, on-pump CABG, isolated valve, combined surgery), duration of aortic clamping (minutes), duration of CPB (minutes), nadir hematocrit during CPB and nadir mean arterial pressure (MAP, mmHg) during surgery. For each patient, the mortality scores (Parsonnet [4], EuroSCORE1 [5]) were calculated. In-ICU and in-hospital lengths of stay, as well as in-hospital mortality rates, were considered. Patients’ 1-year survival rate was established on the basis of post-operative follow-up and/or phone contact.

### Assessment of renal function at baseline and after surgery

Baseline serum creatinine levels were systematically obtained at the time of the preoperative consultation in Anaesthesiology (i.e. 7 days before surgery) and measured by the isotope dilution mass spectrometry traceable Jaffe method from Roche®. GFR was estimated using the Modification of the Diet in Renal Disease (MDRD) formula. Following surgery, AKI was diagnosed within the first 7 days post-surgery. Severity of AKI according to the RIFLE classification was established by using the highest serum creatinine level and/or the lowest urine output. Stages L and E of the RIFLE classification, i.e. complete loss of renal function > 4 weeks or > 3 months, respectively, were not included in the analysis. The management of patients, including the use of diuretics and renal replacement therapy (RRT), was at the discretion of the physicians in charge.

### Statistics

Results were expressed as median and inter-quartile ranges (IQR) and as counts and proportions (%) for qualitative variables. Non parametric Kruskal-Wallis and Wilcoxon tests were used for comparing samples from different groups. Proportions were compared using the chi-squared test for contingency tables. The relationship between patients’ postoperative renal status and a set of covariates was tested using binary logistic regression analysis. Results were expressed in terms of the odds ratio (OR) together with its 95 % CI. Survival data were illustrated using the Kaplan-Meier method, with the Cox proportional hazard (PH) model used to assess the effect of covariates on survival. All results were considered to be significant at the 5 % critical level (p < 0.05). Data analysis was carried out using, Satistica (version 10), SAS (version 9.3 for Windows) and S-PLUS (version 8.1) statistical softwares.

## Results

### Characteristics of the cohort

On the basis of the selection criteria, 443 patients were included during the study period. Baseline demographic, clinical, biological and operative characteristics of this cohort are summarized in Table 1. Proportion of male patients was predominant (70.0 %). The median age was 69 years [60–76]. Preoperative diagnostic of hypertension was present in 81.0 % of the patients. Most patients (76.7 %) had a normal renal function before surgery. Patients presenting with preoperative chronic kidney disease (CKD) were significantly older than individuals with normal kidney function (74-y vs 66-y, p = 0.0001), and included a higher proportion of females (41.7 % vs 26.5 %, p < 0.0001). The median mortality risks assessed by Parsonnet and EuroSCORE1 scores were 6.30 (3.10-13.6) and 4.00 (2.10-7.50), respectively, for the entire cohort. Two hundred and forty patients (54.2 %) were operated by CABG alone and 58 (13.1 %) of them were off-pump procedures.

**Table 1:** Baseline, clinical and operative characteristics (N = 443)

Variable	
Age (years)	69.0 (60.0 - 76.0)
Gender (Female) (%)	133 (30.0)
BMI (kg/m^2^)	26.2 (23.7 - 29.4)
Hypertension (%)	351 (81.0)
COPD (%)	84 (19.0)
Angina pectoris (%)	197 (44.5)
Myocardial infarction previous surgery (%)	116 (26.2)
Diabete mellitus (%)	100 (22.6)
Emergency (%)	12 (2.70)
Preoperative GFR (mL/min)	78.0 (64.0 - 92.0)
Preoperative hemoglobin (g/dL)	13.9 (12.6 - 14.8)
LVEF < 60 % (%)	114 (25.7)
Logistic Parsonnet (%)	6.30 (3.10 - 13.6)
Logistic EuroSCORE1 (%)	4.00 (2.10 - 7.50)
Surgical delay (days)	21.0 (7.00 - 42.0)
Type of surgery:	
OPCABG (%)	58 (13.1)
CABG (%)	182 (41.1)
Valve surgery (%)	148 (33.4)
Valve and CABG (%)	55 (12.4)
CPB time (min.)^*^	87.0 (73.0 - 104.0)
Aortic cross clamp time (min.)^*^	55.0 (41.0 - 69.0)
Nadir hematocrit (%)	21.0 (19.0 - 24.0)
Nadir MAP during surgery (mmHg)	45.0 (40.0 - 55.0)
Days in ICU	2.0 (2 - 4)
Days in hospital	12.0 (10 - 18)

Data are expressed as median (25-75th percentiles) or proportion of patients in %

^*^Data are calculated as median (25-75th percentiles) only for cases with cardiopulmonary bypass (n = 385)

*BMI*: body mass index; *COPD*: chronic obstructive pulmonary disease; *GFR*: glomerular filtration rate; *LVEF*: left ventricular ejection fraction; surgical delay: delay between diagnostic coronarography and surgery; *OPCABG*: off-pump coronary bypass graft; *CABG*: coronary artery bypass graft with cardiopulmonary bypass; *Valve surgery*: aortic valve replacement or mitral valve repair/replacement surgery; *Valve and CABG*: aortic valve replacement or mitral valve repair/replacement surgery combined with coronary artery bypass graft; *CPB*: cardiopulmonary bypass; *MAP*: mean arterial pressure.^*^: n = 385; *ICU*: intensive care unit

### Incidence of AKI post cardiac surgery using combined or isolated criteria of the RIFLE classfication

Among the cohort of 443 patients, 221 (49.9 %) developed AKI within the week following surgery according to the conventional RIFLE classification. Eighty patients (36.2 %) reached stage RISK, 114 (51.6 %) stage INJURY and 27 (12.2 %) stage FAILURE. Stages L and E of the RIFLE classification were not included in our analysis. Of note, no patient actually reached L and E stages. Table 2 summarizes the clinical and operative features of the patients with or without postoperative AKI. Patients developing AKI were significantly older and heavier, with a lower preoperative GFR than non-AKI patients. Their logistic Parsonnet and EuroSCORE1 scores, as well as their program of surgery, were significantly worse. Of note, 8 patients (1.08 % of the cohort) required renal replacement therapy (RRT). Only 2 of them recovered normal renal function, while the other 6 died during their in-hospital stay.

**Table 2:** Distribution of preoperative and operative data according to occurence or not of AKI allocated to RIFLE (Risk, Injury and Failure grade) and by the serum creatinine (SCr) or urine output (UO) criteria AKI

Variable	No AKI	AKI	*p*-value	AKI SCr	AKI UO	*p*-value
	(N = 222)	(N = 221)		(N = 43)	(N = 178)	
Age (years)	65.0 (59.0 - 74.0)	71.0 (63.0 - 73.0)	< 0.001	71.0 (60.0 - 77.0)	71.0 (63.0 - 77.0)	0.98
Gender (Female) (%)	61 (27.5)	72 (32.5)	0.21	12 (27.9)	60 (33.7)	0.47
BMI (kg/m²)	25.6 (22.9 - 28.3)	26.9 (24.3 - 30.5)	< 0.001	28.3 (24.8 - 30.9)	26.6 (24.4 - 30.4)	0.25
Hypertension (%)	178 (80.2)	173 (78.3)	0.62	40 (93.0)	133 (74.7)	< 0.001
COPD (%)	39 (17.6)	45 (20.4)	0.45	14 (32.6)	31 (17.4)	0.03
Angina pectoris (%)	106 (47.7)	91 (41.2)	0.16	16 (37.2)	75 (42.1)	0.55
MI previous surgery (%)	67 (30.2)	49 (22.2)	0.06	10 (23.3)	39 (21.9)	0.85
Diabetes mellitus (%)	44 (19.8)	56 (25.3)	0.16	14 (32.6)	42 (23.6)	0.22
Emergency (%)	8 (3.60)	4 (1.81)	0.24	0 (0.00)	4 (2.25)	0.32
Preoperative GFR						
(mL/min)	83.0 (73 - 97)	72.0 (56 - 89)	< 0.001	67.0 (49 - 78)	73.5 (56 - 89)	0.06
Preoperative						
Hemoglobin (g/dL)	14.0 (12.7 - 14.9)	13.5 (12.4 - 14.7)	0.10	12.9 (11.5 - 14.6)	13.8 (12.5 - 14.8)	0.06
LVEF < 60% (%)	58 (26.1)	56 (25.3)	0.85	13 (30.2)	43 (24.2)	0.41
Logistic						
Parsonnet (%)	5.12 (2.65 - 10.8)	7.60 (4.05 - 17.3)	< 0.001	8.41 (5.16 - 15.9)	7.47 (4.00 - 18.7)	0.71
Logistic						
EuroSCORE1 (%)	3.51 (2.01 - 6.48)	4.79 (2.28 - 8.58)	0.004	5.29 (2.73 - 11.0)	4.65 (2.21 - 8.44)	0.40
Surgical delay (days)	20.0 (6 - 39)	22.0 (8 - 45)	0.18	17.0 (5 - 35)	27.0 (8 - 48)	0.02
Type of surgery						
OPCABG (%)	37 (16.7)	21 (9.50)	< 0.001	6 (14.0)	15 (8.43)	0.13
CABG (%)	102 (45.9)	80 (36.1)		13 (30.2)	67 (37.6)	
Valve surgery (%)	74 (33.3)	74 (33.5)		19 (44.2)	55 (30.9)	
Valve and CABG (%)	9 (4.05)	46 (20.8)		5 (11.6)	41 (23.0)	
CPB time (min.)^*^	85.0 (70 - 100)	90.0 (75.5 - 108.0)	0.02	89.0 (81.0 - 100.0)	90.0 (74.0 - 109.0)	0.80
Aortic cross						
Clamp time (min.)^*^	52.0 (39.0 - 64.0)	58.0 (44.0 - 75.0)	0.003	58.0 (45.0 - 71.0)	58.5 (43.5 - 76.0)	0.83
Nadir hematocrit (%)	22.0 (19 - 24)	20.0 (19 - 24)	0.06	20.0 (19.0 - 24.0)	20.0 (19.0 - 24.0)	0.83
Nadir MAP						
During surgery (mmHg)	45 (40 -55)	45 (40 -55)	0.57	45 (40 -55)	45 (40 -50)	0.50
Days in ICU	2.0 (2 - 3)	2.0 (2 -4)	< 0.001	3.0 (2 - 6)	2.0 (2 - 4)	0.07
Days in hospital	12.0 (10 - 16)	13.0 (10 - 18)	0.55	14.0 (11 - 22)	12.0 (10 - 18)	0.07

Data are expressed as median (25-75th percentiles) or proportion of patients in %

^*^Data are calculated as median (25-75th percentiles) only for cases with cardiopulmonary bypass (n = 385)

*BMI*: body mass index; *COPD*: chronic obstructive pulmonary disease; *MI*: myocardial infarction; *GFR*: glomerular filtration rate; *LVEF*: left ventricular ejection fraction; Surgical delay: delay between diagnostic coronarography and surgery; *OPCABG*: off-pump coronary artery bypass graft; *CABG*: Coronary artery bypass graft with cardiopulmonary bypass; *Valve surgery*: aortic valve replacement or mitral valve repair/replacement surgery; *Valve and CABG*: aortic valve replacement or mitral valve repair/replacement surgery combined with coronary artery bypass graft; CPB: cardiopulmonary bypass; MAP: mean arterial pressure; ICU: intensive care unit

The RIFLE criterion SCr only identified 9.7 % of patients developing postoperative AKI, whereas the UO criterion diagnosed AKI in 40.2 % of cases (Table 2). Patients developing AKI_UO_ presented a significantly lower rate of preoperative hypertension (p < 0.001) and COPD (p < 0.05) than patients developing AKI_SCr_. Preoperative GFR and hemoglobin levels were higher in the AKI_UO_ than AKI_SCr_ patients. The delay between cardiologic exploration and heart surgery was also significantly longer in the AKI_UO_ group (p < 0.05).

### Influence of AKI definition on the length of in-hospital and ICU stays and mortality

On the basis of the full RIFLE classification, the duration of the ICU stay was shorter for patients who did not develop AKI after surgery than for AKI patients (2 [2 - 3] vs 3 [2 - 4] days; p < 0.001). However, no significant difference was observed regarding the length of in-hospital stay. The ICU period significantly increased with the degree of AKI severity based on the RIFLE stages: 2.0 (2–3) days for AKI RISK (n = 80), 3.0 (2 - 5) days for AKI INJURY (n = 114), and 5.0 (3 - 12) days for AKI FAILURE (n = 27); p < 0.001. When comparing either criterion of the RIFLE classification as the sole indicator of AKI, the duration of ICU stay tended to be slightly longer in AKI_SCr_ as compared to AKI_UO_ (3.0 [2 - 6] vs 3.0 [2 - 4]; p = 0.07). Concerning mortality, 14 patients (3.16 %) died during their in-hospital stay. Causes of in-hospital mortality were: cardiogenic shock (n = 5), sepsis (n = 4), hemorrhagic shock (n = 3), stroke (n = 1), and aortic dissection (n = 1).

**Fig. 1:**
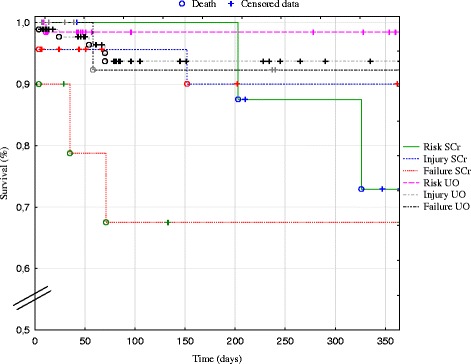
Survival post cardiac surgery according to acute kidney injury severity. Kaplan-Meier survival curve post cardiac surgery in patients with increasing RIFLE stages of acute kidney injury on the basis of elevated serum creatinine (SCr) or decreased urine output (UO)

**Fig. 2:**
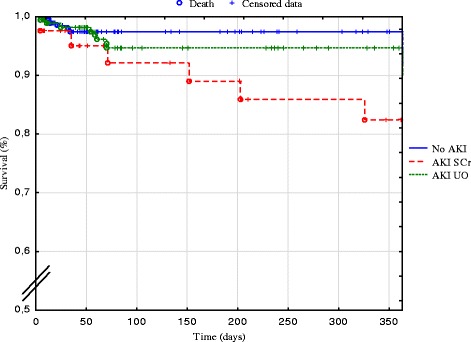
Survival post cardiac surgery according to AKI status. Kaplan-Meier survival curve post cardiac surgery in patients with no acute kidney injury (AKI, blue line), AKI with elevated serum creatinine (SCr, dotted red line) and AKI with decreased urine output (UO, dotted green line)

At one year post surgery, the follow-up was available for 364 patients of the cohort (82.2 %), and a sum of 20 deceased patients was recorded. Preoperative characteristics which were significantly associated with an increased risk for 1-year mortality included: age, COPD, CKD, anaemia, LEVF < 60 % and surgical delay (Table 3). The complexity of surgery, including the nadir of per-operative haematocrit, was also identified as a significant risk factor for death at 1-year *post* cardiac surgery. Postoperative AKI was also associated with an increased risk for 1-year mortality (p < 0.05, Table 3), which was further accentuated upon AKI severity score (p < 0.01, Fig. 1). One-year mortality rate was significantly higher in AKI_SCr_ patients in comparison to AKI_UO_ group (p < 0.05, Fig. 2).

**Table 3:** Distribution of preoperative and operative data according to status (death or alive) at one year after surgery

Variable	Death	Alive	*p*-value
	(N = 20)	(N = 344)	
Age (years)	77.0 (69.5 - 79.5)	68.5 (60.0 - 75.0)	0.004
Gender (Female) (%)	9 (45.0)	94 (27.3)	0.89
BMI (kg/m^2^)	24.5 (22.6 - 27.9)	26.2 (23.6 - 29.3)	0.28
Hypertension (%)	7 (35.0)	56 (16.3)	0.74
COPD (%)	7 (35.0)	56 (16.3)	0.03
Angina pectoris (%)	9 (45.0)	149 (43.3)	0.88
MI previous surgery (%)	3 (15.0)	87 (25.3)	0.30
Diabetes (%)	4 (20.0)	80 (23.3)	0.74
Emergency (%)	1 (5.00)	8 (2.33)	0.45
Preoperative GFR (mL/min)	60.0 (43.0 - 75.0)	79.5 (65.0 - 93.0)	0.002
Preoperative hemoglobin (g/dL)	11.4 (9.9 - 13.0)	13.9 (12.6 - 14.9)	< 0.001
LVEF < 60 %	10 (50.0)	82 (23.9)	0.008
Surgical delay (days)	14.0 (4.0 - 19.0)	22.0 (8.0 - 45.0)	0.008
Type of surgery			
OPCABG (%)	1 (5.00)	42 (12.2)	0.41
CABG (%)	6 (30.0)	142 (41.3)	
Valve surgery (%)	9 (45.0)	116 (33.7)	
Valve and CABG (%)	4 (20.0)	44 (12.8)	
CPB time (min.)^*^	89.5 (79.0 - 110.0)	85.0 (66.5 - 102.5)	0.19
Aortic cross clamp time (min.)^*^	59.5 (44.5 - 78.0)	51.0 (35.0 - 67.0)	0.13
Nadir hematocrit (%)	19.0 (18.0 -19.0)	21.0 (19.0 - 24.0)	< 0.001
Nadir MAP during surgery (mmHg)	45.0 (40–53)	45.0 (40–55)	0.68
Postoperative AKI (%)	15 (75.0)	175 (50.9)	0.04

Data are expressed as median (25-75th percentiles) or proportion of patients in %

^*^Data are calculated as median (25-75th percentiles) only for cases with cardiopulmonary bypass (n=385)

*BMI*: body mass index; *COPD*: chronic obstructive pulmonary disease; *MI*: myocardial infarction; *GFR*: glomerular filtration rate; *LVEF*: left ventricular ejection fraction; Surgical delay: delay between diagnostic coronarography and surgery; *OBCABG*: off-pump coronary artery bypass graft; *CABG*: Coronary artery bypass graft with cardiopulmonary bypass; *Valve surgery*: aortic valve replacement or mitral valve repair/replacement surgery; *Valve and CABG*: aortic valve replacement or mitral valve repair/replacement surgery combined with coronary artery bypass graft; *CPB*: cardiopulmonary bypass; *MAP*: mean arterial pressure

### Predictive pre- and per-operative risk factors for AKI

In univariate analysis adjusted for preoperative CKD, six variables were positively associated with the occurrence of AKI defined upon the conventional RIFLE classification: age (OR = 1.02 [IC95 % 1.01-1.04], p = 0.016), BMI (OR = 1.08 [IC95 % 1.03-1.13], p = 0.001), logistical Parsonnet score (OR = 1.05 [IC95 % 1.03-1.09], p < 0.0001), combined surgery (OR = 6.26 [IC95 % 2.88-13.6], p < 0.0001), duration of aortic clamping (OR = 1.01 [IC95 % 1.01-1.02], p = 0.007) and duration of CPB (OR = 1.02 [IC95 % 1.01-1.03], p = 0.001). In multivariate setting, the occurrence of AKI increased with BMI (OR = 1.12 [IC95 1.06-1.18], p < 0.001), the type of surgery (combined surgery (OR = 10.03 [IC95: 3.11-33.9], p < 0.001)), and the lower preoperative GFR (OR = 0.98 [IC95: 0.97-0.99], p = 0.004) (Table 4).

**Table 4:** Multivariate binary logistic regression of AKI development according to perioperative factors

	OR	95 % CI	*p-value*
Age (years)	1.02	1.00 - 1.04	0.11
Gender (Female vs Male)	0.91	0.52 - 1.59	0.73
BMI (kg/m^2^)	1.12	1.06 - 1.18	< 0.001
Hypertension (Yes vs No)	0.75	0.44 - 1.28	0.28
COPD (Yes vs No)	1.18	0.67 - 2.09	0.56
Angina pectoris (Yes vs No)	0.70	0.64 - 1.77	0.80
MI previous surgery (Yes vs No)	0.74	0.44 - 1.27	0.28
Diabetes mellitus (Yes vs No)	1.11	0.64 - 1.91	0.72
Emergency (Yes vs No)	0.39	0.09 - 1.72	0.21
Preoperative GFR (mL/min.)	0.98	0.97 - 0.99	0.004
Preoperative hemoglobin (g/dL)	0.02	0.87 - 1.19	0.84
LVEF <60 % (Yes vs No)	1.01	0.60 - 1.71	0.97
Surgical delay (days)	1.00	1.00 - 1.01	0.47
Type of surgery:			
Valve (vs CABG)	1.56	0.70 - 3.51	0.28
Valve and CABG (vs CABG)	10.3	3.11 - 33.9	< 0.001
CPB time (min.)^*^	1.01	1.00 - 1.02	0.17
Aortic cross clamp time (min.)^*^	0.99	0.96 - 1.01	0.23
Nadir hematocrit (%)	1.01	0.94 - 1.08	0.79
Nadir MAP during surgery (mmHg)	0.99	0.96 - 1.01	0.36

*OR*: odds ratio ± 95 % confidence interval; *BMI*: body mass index; *COPD*: chronic obstructive pulmonary disease; *MI*: myocardial infarction; *GFR*: glomerular filtration rate; *LVEF*: left ventricular ejection fraction; Surgical delay: delay between diagnostic coronarography and surgery; *CABG*: coronary artery bypass graft with cardiopulmonary bypass; *Valve surgery*: aortic valve replacement or mitral valve repair/replacement surgery; *Valve and CABG*: aortic valve replacement or mitral valve repair/replacement surgery combined with coronary artery bypass graft; *CPB*: cardiopulmonary bypass; *MAP*: mean arterial pressure; *, n=385

## Discussion

The present retrospective monocentric study includes 443 patients undergoing elective or emergent cardiac surgery, and investigates the incidence and outcomes of postoperative AKI, as well as the pre- and per-operative risk factors for AKI. The incidence and outcomes of AKI vary according to its definition. In the present study, we first used the conventional 2004 RIFLE classification which is based on the elevation of SCr and the decrease of UO [6]. This classification has been approved for cardiac surgery [1, 7–9]. In an original approach, we compared AKI incidence and outcomes according to SCr or UO criteria. We observed a 7-day incidence of AKI reaching 50 % by taking into account both RIFLE criteria. This percentage of postoperative AKI is rather high, as compared to previous reports. This may be partly explained by the use of all daily values of SCr and UO for each patient over the 7-day period following surgery. In a similar retrospective study, D’Onofrio et al. reported an incidence of postoperative AKI of 23.5 % [7]. These authors only tracked the single variation in SCr during the length of hospitalisation. Conversely, by using the complete RIFLE classification, Hobson et al. reported an incidence of AKI reaching 43.0 % after cardiac surgery, which corresponds with our observations [1]. Interestingly, each component of RIFLE, i.e. SCr or UO, differentially contributes to AKI incidence and outcomes, as previously illustrated by McIlroy et al. [10] and Wlodzimirow, et al. [11]. In our series, the elevation of SCr was observed in 9.7 %, whereas a decreased UO was noted in 40.2 %. Such a 4-fold increase in AKI incidence when the UO criterion is taken into account provides cause for concern regarding its validity and the true significance of diagnosed AKI_UO_. To apply the SCr criteria of RIFLE, information on prior renal function is needed. When a pre-ICU admission SCr is not available, RIFLE group suggests that the baseline SCr be estimated from the MDRD formula [3]. Still, mathematical estimations of baseline SCr have been associated with both over- or under-diagnosis of AKI [12, 13]. Here, baseline SCr systematically corresponded with its value 7 days before surgery. Patients developing AKI_SCr_ showed a significantly higher preoperative morbidity, including hypertension, COPD, CKD and anaemia, than AKI_UO_ patients (Table 2). Moreover, 1-year mortality rate was significantly higher in the case of AKI_SCr_ compared to AKI_UO_ (Fig. 1). Systematic reviews investigating the validity of RIFLE classification in general ICU patients concluded that the mortality risk was higher in the absence of UO criterion in the AKI definition [14, 15]. Still, discarding the UO criterion has been suggested to delay the diagnosis of AKI, thereby increasing the mortality in general ICU patients [11]. In the particular settings of patients undergoing cardiac surgery, oliguria has also been associated with an increased incidence of AKI, but with a reduced discriminant utility for mortality in comparison to elevated SCr [10, 16]. As a whole, the inclusion of UO consensus criterion causes a questionable impact on the overestimated incidence of AKI after cardiac surgery.

The pathophysiology of postoperative AKI is multifactorial [17]. In our cohort, patients developing *post* surgery AKI were older and more fragile according to the Parsonnet and EuroSCORE1 scores, and presented with a higher BMI. Furthermore, increased BMI is significantly associated with DM (29.0 [25.8 - 31.6] vs 25.7 [23.1 - 28.3], p < 0.001) and hypertension (26.4 [23.9 - 29.9] vs 25.7 [23.0 - 27.8], p < 0.02). These risk factors were further highlighted by our multivariate model (Table 4). Pre-operative CKD reflected by a lower GFR before surgery was also associated with an increased risk for AKI. We estimated the pre-operative GFR using the MDRD formula based on serum creatinine concentration 7 days before surgery [18]. Most patients (76.7 %) showed a GFR within the normal range, i.e. > 60 ml/min. Patients presenting with impaired kidney function before surgery were significantly older (74 [68 - 78] years) than patients with GFR > 60 mL/min (66 [59 - 74] years, p < 0.001). This represents a selection bias. Indeed, given the impact of age in the multivariate logistical regression Parsonnet model, the predicted mortality rate at 30 days *post* surgery was higher in the CKD group compared to the rest of the cohort. The same pattern was observed with the logistical model EuroSCORE1 [5]. Similarly, we observed a lower 1-year survival probability in patients with a pre-operative GFR <60 mL/min [19].

The type and complexity of cardiac surgery was identified as an independent predictor of post-operative AKI upon our multivariate model (Table 3). At our institution, the choice between Off-pump and On-pump procedures essentially depends on the technical feasibility of myocardial revascularisation on the basis of coronarography images. In the present cohort, the types of surgeries, with or without CPB, are not distributed homogeneously, with more patients with normal renal function (15.3 %) being operated on using the Off-pump technique compared to CKD patients (5.83 %). Still, we did not observe a significantly higher incidence of AKI in On-pump versus Off-pump groups, in contrast to previous reports [20–22]. Patients with normal pre-operative renal function were mostly operated for isolated CABG, whereas only a minority (10.0 %) underwent combined surgery. Conversely, the distribution of surgery types appears more balanced in the CKD group, with 39.8 % of patients operated for CABG or isolated valve and 20.4 % for combined surgery. Such an imbalance between non-CKD and CKD patients may represent an additional selection bias in our analysis since the type of surgery influences the duration of aortic clamping and CPB, which in turn may favor AKI development.

In the present cohort, post-operative AKI was associated with a significantly longer stay in the ICU, with no impact on the entire in-hospital period. Moreover, the ICU period significantly increased with the degree of AKI severity based on the RIFLE stages. Elevation of SCr appeared to be associated with a prolonged ICU stay in comparison to UO, although this observation did not reach statistical significance (p = 0.07). Requirement for RRT was associated with a dramatic increase in the in-hospital mortality (75.5 %), as was previously reported [23–25].

The present study has several limitations, including its retrospective and monocentric design with a limited number of patients. Secondly, the majority of surgical interventions were planned (97.3 %), which limits the extension of the observations to emergency surgeries. Indeed, the need for nephrotoxic contrast medium at the time of preoperative coronarography may precipitate AKI development in cases of early cardiac surgery [26, 27]. Thirdly, the priming solution of CPB circuit most often consists of Volulyte 6 % (4th generation balanced hydroxyethyl starch). Recently, the use of such a starch has been associated with an increased incidence of AKI, as well as with postoperative bleeding [28]. Volulyte 6 % solution was chosen for CPB priming in order to reduce the risk for anaphylactoid shocks observed with some colloids or gelatins. New generation starches appear to be less nephrotoxic, provided that the perfusion is not extended beyond 24 h [29–31]. The optimal priming solution for CPB circuits remains a matter of debate.

## Conclusions

The use of the conventional RIFLE classification in a cohort of 443 patients undergoing cardiac surgery found an incidence of AKI reaching 50 %. When each RIFLE criterion was considered individually, a significant discrepancy was found between AKI_SCr_ and AKI_UO_ regarding AKI incidence and outcomes. AKI_SCr_ most often occurs in patients with a high-risk pre-operative profile, and is associated with an increased 1-year mortality compared to AKI_UO_. In line with previous reports, our present study urges the need for appropriately powered multi-centric follow-up trials to clarify the actual utility of UO as a criterion for AKI diagnosis and management in patients undergoing cardiac surgery.

## Abbreviations

AKI: Acute kidney injury
CABG: Coronary artery bypass graft
CKD: Chronic kidney disease
COPD: Chronic obstructive pulmonary disease
CPB: Cardioplumonary bypass
GFR: Glomerular filtration rate
ICU: Intensive care unit
MAP: Mean arterial pressure
MDRD: Modified of the died in renal disease
OPCAB: Off-pump coronary artery bypass graft
RIFLE: Risk, injury, failure, loss of function, end stage kidney disease
RRT: Renal replacement therapy
SCr: Serum creatinine
UO: Urine output
LVEF: Left ventricular ejection fraction
